# A semi-analytical solution to organic contaminants transport through composite liners considering a single crack in CCL

**DOI:** 10.1007/s11356-021-18171-1

**Published:** 2022-01-27

**Authors:** Haijian Xie, Hao Ding, Huaxiang Yan, Dandi Yang, Zhanghua Lou, Zhanhong Qiu, Yun Chen

**Affiliations:** 1 Center for Balance Architecture, Zhejiang University, 148 Tianmushan Rd., Hangzhou, 310007 China; 2 College of Civil Engineering and Architecture, Zhejiang University, 866 Yuhangtang Rd., Hangzhou, 310058 China; 3 Ocean College, Zhejiang University, Zheda Rd., Zhoushan, 316021 China; 4 Department of Mechanical, Aerospace and Civil Engineering, School of Engineering, The University of Manchester, Manchester, M13 9PL UK; 5 School of Civil Engineering and Architecture, Taizhou University, Taizhou, 318000 China; 6 The Architectural Design and Research Institute of Zhejiang University Co. Ltd., 148 Tianmushan Rd., Hangzhou, 310058 China

**Keywords:** Landfill composite liner, Organic contaminant, Cracks, Semi-analytical solution

## Abstract

Compacted clay liners (CCLs) are extensively used as engineering barriers for groundwater and soil pollution. The existence of cracks/fractures in CCL caused by thermally induced shrinkage is reported to importantly damage the performance of the CCL. An analytical model is developed to study the effects of the cracks/fractures on the migration of organic contaminants through a composite liner system. Laplace transformation and Laplace inversion using the Stehfest method are adopted to derive the analytical solution, which is validated by the experimental data. The existence of crack shows a significant impact on the breakthrough curve and bottom flux of organic contaminants. Increasing the crack width from 1 to 25 mm results in an enhancement of contaminant bottom concentration by a factor of 280. Increasing the adsorption factor and degradation rate of contaminants can effectively improve the performance of the composite liner with cracks. The effects of degradation of contaminants on the breakthrough curve are found to be more significant for the case with a larger retardation factor. This may be due to the fact that increasing the retardation factor can significantly slow down the transport of contaminants, which may indirectly create a longer period for the degradation of contaminants.

## Introduction

Landfilling is currently one of the main methods of disposal of municipal waste. The sanitary volume of municipal domestic waste in mainland China was reported to be 109.480 million tons with a landfill disposal rate of 45.23% (NBSC 2019). Even at regular landfills, leachate with complex components can still form during long-term operation and pose a threat to the environment. There were 1259 organic compounds detected from rivers and wells around the landfill reported by the U.S. Environmental Protection Agency (USEPA) in 1976 (Shackelford and Keith 1976; Kjeldsen et al. 2002). Öman and Junestedt 2008) screened leachate samples from 12 Swedish municipal landfill sites for 400 parameters and compounds where more than 90 organic and metal–organic compounds were detected. Masoner et al. (2014) sampled fresh leachate from 19 landfills across the USA during 2011. A total of 129 out of 202 contaminants of emerging concern (CECs) were detected, including 62 prescription pharmaceuticals, 23 industrial chemicals, 18 nonprescription pharmaceuticals, 16 household chemicals, 6 steroid hormones, and 4 plant/animal sterols. Most of them are organic contaminants.

It is important to study organic contaminant transport as they can cause very serious harm to humans and various organisms in nature, including serious ecological hazards and risks (Batt et al. 2017; Peng et al. 2018; Koual et al. 2019; Espinosa-Reyes et al. 2019). For example, polychlorinated biphenyl (PCB) can lead to neurological, endocrine, genetic, and systemic adverse effects in the human body (Hens and Hens 2018). Polycyclic aromatic hydrocarbons (PAHs) are associated with risks to human health (e.g., carcinogenesis) (Sampaio et al. 2021). Some phenolic compounds are known to be endocrine-disrupting compounds (EDCs), which have a detrimental effect on the endocrine system, such as 2,4,6-trichlorophenol (2,4,6-TCP) (Chen et al. 2021).

Predictive capability for a quantitative assessment of the contaminant transport processes is necessary for designing an effective and well-operational liner system. Although numerical models are generally used to study the contaminant transport problems, some complex numerical simulations are very time-consuming due to the large amount of data required to support the numerical models. Analytical solutions are therefore critically important for understanding many scientific phenomena (e.g., contaminant transport, heat transfer, and deformation), even though simplifications may be made to derive them (Yan et al. 2021c). Especially, analytical solutions play a unique role in verifying many new numerical methods. For example, the simplified analytical solutions, however, allow assessment of the sensitivity to various instabilities involved in the transport of contaminants in various landfill barrier systems and verification of the results of complex analyses (Rowe et al. 2004; Dejam 2019; Feng et al. 2020). For these reasons, analytical solutions have been extensively derived in recent years for investigating the performance of composite liner under different conditions (Xie et al. 2013, 2015a, b, 2016; Rowe and Abdelatty 2013; Wu et al. 2017; Feng et al. 2019; Yan et al. 2021a, b). The existing analytical models mentioned above were developed based on the assumption of intact clay liner without fractures/cracks. However, the cracks can be easily induced in the liner system, such as shrinkage cracks due to tension generated during drying (Inci 2008; Tang et al. 2008b), thermal cracks due to thermal stress changes in the soil material (Tang et al. 2008a), tensile cracks due to overburden pressure changes (Wu et al. 2012), and fracture cracks due to fracture loads such as external loads and internal cyclic loads (Pal et al. 2009; Wei et al. 2020).

Numerous studies and experimental observations have shown that the formation of cracks in the clay barrier provides a preferential flow for contaminants (Omidi et al. 1996; Rayhani et al. 2007; Li et al. 2016, 2018). The cracks reported from the field observations can reach 15–30 mm in width and 2 m in depth (Ritchie and Adams 1974; Basnett and Brungard 1992; Omidi et al. 1996). The mechanisms for clay desiccation processes have been mainly investigated by using numerical approaches, including the finite element method (FEM) (Trabelsi et al. 2012; Hirobe and Oguni 2017) and the discrete element method (DEM) (Sima et al. 2014; Wei et al. 2020). Studies have also focused on developing analytical models for investigating solute transport in porous media with cracks. An analytical model of solute transport through a single fracture was first proposed by Tang et al. (1981). The model was then extended by Roubinet et al. (2012) to investigate the influences of transverse dispersion in the fracture and longitudinal diffusion in the matrix on solute transport. Other modelling efforts have focused on reactive transport (Zhu et al. 2016), advective-dominated systems (Birkhölzer et al. 1993; Odling and Roden 1997; Houseworth et al. 2013), fracture networks (Cvetkovic and Frampton 2012; Haddad et al. 2012). The above studies were designed for investigating the contaminant transport through fractured rock and other environmental groundwater contaminant problems (e.g., nuclear waste disposal and contaminant removal from the fractured rocks) (Zhu et al. 2016). However, the composite liners of landfills generally consist of CCL and geomembrane (GMB), which is placed on the CCL to inhibit the transport through advection. Additionally, the cracks in the clay liner can create pathways for contaminant transport, which will decrease the performance of clay barriers used for waste isolation (Wan et al. 2018; DeCarlo and Shokri 2014). The traditional diffusion model (e.g., Xie et al. 2013; Rowe and Abdelatty 2013; Wu et al. 2017; Feng et al. 2019; Yan et al. 2021a, b) may lead to overestimation of the performance of composite liner with cracked clay.

The aim of this article is to address the issue in relation to the extent of the effects of cracks in the clay liner on the overall transport of contaminants in a composite liner system by developing a new analytical solution. The developed analytical model can be used to investigate the breakthrough of the organic contaminant through composite liners with cracks or design a more conservative barrier system. The effects of degradation of organic contaminants, the porosity of the CCL, crack width, and the partition coefficient of organic contaminants in GMB are investigated. Additionally, the present analytical solution may be an extension for investigating nuclear waste storage facilities, groundwater pollution problems, and shale gas extraction in a fractured system.

The paper is organised as follows. The next section presents the mathematical model development and basic assumptions for contaminant migration in composite liners with cracks. The third section shows the analytical solution to the problem, while the fourth section presents the validation of the proposed analytical solution against a set of experimental data. Results and discussions presents the results of parametric studies of the effects of several key factors (e.g., crack width, degradation and adsorption of contaminants, and porosity of clay) on the overall transport behaviour of contaminants. Conclusions from the work are given in the last section.

## Mathematical model

**Fig. 1:**
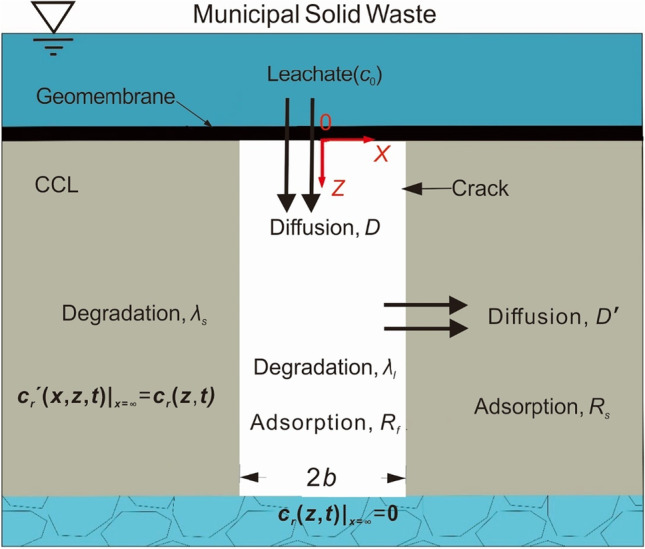
The conceptual model of contaminant transport through a cracked composite liner system

In this study, we consider a composite liner consisting of intact GMB and CCL with a regular strip crack of 2*b* width and infinite depth in the CCL. It should be noted that using an infinite depth of composite liner may result in conservative predictions of contaminant transport (Foose 2002) and facilitate the development of analytical models. The GMB is placed on the CCL. The source of leachate contaminant is assumed to be constant. The organic contaminant in the leachate may transport through the GMB by diffusion and then enter the crack. The contaminant transport mechanisms in CCL include diffusion, adsorption on solid particles, and degradation of organic contaminants. The origin of the coordinate axis is the opening of the crack, and the *z*-axis is positive downward (as shown in Fig. 1).

The basic assumptions of the present model are as follows: (i) the width of the crack is much smaller than its length; and therefore, the width is negligible with respect to the length. (ii) In the crack, the contaminants are fully diffused and dispersed in the lateral direction, and complete mixing in the lateral direction is maintained throughout the flow process; (iii) the CCL is isotropic and homogeneous; (iv) the transport of contaminants along the crack direction is much faster than that in the matrix of CCL; and (v) adsorption is a linear and equilibrium process.

### Governing equation of organic contaminant transport

By using the total balance of contaminants in the crack, we are able to establish the following governing equation (Tang et al. 1981; Xie et al. 2019):

1 $$\begin{array}{cc}\frac{\partial {c}_{r}(z,t)}{\partial t}+\frac{1}{b}\frac{\partial s}{\partial t}=D\frac{{\partial }^{2}{c}_{r}(z,t)}{\partial {z}^{2}}-{\lambda }_{l}\left[{c}_{r}\left(z,t\right)-\frac{s}{b}\right]-\frac{q}{b}& 0\le z\le \infty \end{array}$$

and

2 $${\lambda }_{l}=(\mathit{ln}2)/{t}_{f,1/2}$$

where *z* is a coordinate along the crack axis, L; *t* is time, T; *c*_*r*_(*z*, *t*) is the concentration of contaminants in cracks, M/L^3^; *s* is the mass of pollutants absorbed on CCL per unit length of crack surface, M/L^2^; *b* is half the width of the crack, L; *λ*_*l*_ is first-order degradation rate coefficient of organic contaminants, 1/T; *t*_*f*,1/2_ is the half-life of organic contaminants in cracks, T; *q* is diffusive flux perpendicular to the crack axis, M/L^2^T; *D* is the diffusion coefficient of organic contaminants in cracks, L^2^/T.

Assuming that the absorption process of contaminants at the crack surface obeys linear equilibrium, the dissolution and absorption processes can be expressed by the following two equations (Tang et al. 1981):

3a $$s=\frac{ds}{dc}{c}_{r}(z,t)={\kappa }_{f}{c}_{r}(z,t)$$

3b $$\frac{\partial s}{\partial t}=\frac{\partial s}{\partial {c}_{r}(z,t)}\frac{\partial {c}_{r}(z,t)}{\partial t}={\kappa }_{f}\frac{\partial {c}_{r}(z,t)}{\partial t}$$

where ${\kappa }_{f}$ is the distribution coefficient.

By substituting Eqs. (3a) and (3b) into Eq. (1), the governing equation describing the transport of organic contaminants in the crack is transformed into (Tang et al. 1981)

4 $$\begin{array}{cc}{R}_{f}\frac{\partial {c}_{r}(z,t)}{\partial t}=D\frac{{\partial }^{2}{c}_{r}(z,t)}{\partial {z}^{2}}-{\lambda }_{l}{R}_{f}{c}_{r}\left(z,t\right)-\frac{q}{b}& 0\le z\le \infty \end{array}$$

and

5 $${R}_{f}=1+\frac{{\kappa }_{f}}{b}$$

where ${\text{R}}_{\mathrm{f}}$ is the crack retardation factor.

Similarly, the transport of contaminants in CCL can be obtained by considering the total balance of contaminants per unit width and the diffusion perpendicular to the crack direction (Tang et al. 1981; Xie et al. 2019):

6 $$\frac{\partial {c}_{r}^{\prime}(x, z, t)}{\partial t}+\frac{{\rho }_{b}}{\theta }\frac{\partial {s}^{\prime}}{\partial t}={D}^{\prime}\frac{{\partial }^{2}{c}_{r}^{\prime}(x,z,t)}{\partial {x}^{2}}-{\lambda }_{s}{(c}_{r}^{\prime}\left(x,z,t)+\frac{{\rho }_{b}}{\theta }{s}^{\prime}\right) b \le x \le \infty$$

and

7 $${\lambda }_{s}=(\text{ln}2)/{t}_{s,1/2}$$

where *x* is the coordinate perpendicular to the crack axis, L; *c*′_*r*_(*x*,*z*,*t*) is the concentration of contaminants in solution, M/L^3^; *s*′ is the mass of solute absorbed per unit of solid in CCL, M/L^2^; *ρ*_*b*_ is the density of CCL, M/L^3^; *θ* is the porosity of CCL; *λ*_*s*_ is the first-order degradation rate coefficient of organic contaminants in CCL, 1/T; and *t*_*s*,1/2_ is the half-life of organic contaminants in the matrix of CCL, T.

In the above equation, the effective diffusion coefficient *D*′ is defined as

8 $$D^{\prime}=\tau D$$

where *τ* is the matrix tortuosity (Bear 2013).

A linear equilibrium isotherm adsorption model is used to describe the sorption of contaminant on CCL as follows:

9a $$s^{\prime}=\frac{ds^{\prime}}{d{c}_{r}^{\prime}(x,z,t)}{c}_{r}^{\prime}(x,z,t)={\kappa }_{m}{c}_{r}^{\prime}(x,z,t)$$

9b $$\frac{\partial s^{\prime}}{\partial t}=\frac{ds^{\prime}}{d{c}_{r}^{\prime}(x,z,t)}\frac{\partial {c}_{r}^{\prime}(x,z,t)}{\partial t}={\kappa }_{m}\frac{\partial {c}_{r}^{\prime}(x,z,t)}{\partial t}$$

where ${\kappa }_{m}$ is the distribution coefficient in CCL. The CCL retardation factor can be defined as

10 $${R}_{s}=1+\frac{{\rho }_{b}}{\theta }{\kappa }_{m}$$

Combining Eqs. (6), (9), and (10), the governing equation of CCL can be obtained:

11 $$\frac{\partial {c}_{r}^{\prime}(x,z,t)}{\partial t}-\frac{{D}^{\prime}}{{R}_{s}}\frac{{\partial }^{2}{c}_{r}^{\prime}(x,z,t)}{\partial {x}^{2}}+{\lambda }_{s}{c}_{r}^{\prime}(x,z,t)=0 b \le x \le \infty$$

At this point, let us consider the loss term due to diffusion in Eq. (5). This loss term represents the diffusion flux through the cracked-CCL interface. This flux can be expressed according to Fick’s first law as

12 $${\left.q=-\theta D^{\prime}\frac{\partial {c}_{r}^{\prime}(x,z,t)}{\partial x}\right|}_{x=b}$$

Substituting Eq. (12) into Eq. (5) results in the contaminant migration in the crack:

13 $$\frac{\partial {c}_{r}(z,t)}{\partial t}-\frac{D}{{R}_{f}}\frac{{\partial }^{2}{c}_{r}(z,t)}{\partial {z}^{2}}+{\lambda }_{l}{c}_{r}(z,t)-\frac{\theta {D}^{\prime}}{b{R}_{s}}{\left.\frac{\partial {c}_{r}^{\prime}(x,z,t)}{\partial x}\right|}_{x=b}=0 0 \le z \le \infty$$

### Boundary and initial conditions

The concentration of contaminants inside the GMB can be described by the following equation (El-Zein 2008; Xie et al. 2013):

14 $${D}_{g}\frac{{d}^{2}{c}_{g}(z)}{d{z}^{2}}=0 (-{L}_{g} \le z \le 0)$$

where ${\mathrm{c}}_{\mathrm{g}}{(}{\mathrm{z}}{)}$ is the concentration of organic contaminants in GMB,$\mathrm{M/}{\mathrm{L}}^{3}$; ${D}_{g}$ is the effective diffusion coefficient of organic contaminants in GMB, ${\mathrm{L}}^{2}/\mathrm{T}$; and ${\mathrm{L}}_{\mathrm{g}}$ is the thickness of GMB, L.

Assuming that the concentration of organic contaminants in the leachate on the GMB is a constant (e.g., ${\mathrm{c}}_{0} \, {=} \, {1} \, \text{mg/L}$). A constant contaminant concentration assumption at the top of the GMB is a relatively reasonable boundary condition, which is a relatively conservative estimate of the contaminant transport (Shackelford 1990; Foose 2002). The top boundary condition can be described using the following equation (Rowe 2005):

15 $${c}_{g}(-{L}_{g})={c}_{0}{S}_{gf}$$

where ${\mathrm{S}}_{\text{gf}}$ is partition coefficients between leachate and GMB.

The continuity of contaminant fluxes and concentrations must be satisfied at the interface between the GMB and the CCL. The continuity of the contaminant flux can be described by Fick’s first law (Foose 2002)

16 $${D}_{g}{\left.\frac{d{c}_{g}(z)}{dz}\right|}_{z=0}=\theta {D}^{\prime}{\left.{f}_{r}(z,t)\right|}_{z = 0}$$

where ${\mathrm{f}}_{\mathrm{r}}{(}{\mathrm{z}}{,}{\mathrm{t}}{)}$ can be expressed by the following equation:

17 $${f}_{r}(z,t)=\frac{\partial {c}_{r}(z,t)}{\partial z}$$

Assuming that the contaminants passing through the GMB will enter the crack completely, there should be a continuity of flow and concentration at the interface between the crack and the GMB, and the continuity of concentration at the interface of the GMB and the CCL can be expressed by the following equation (Kalbe et al. 2002; Chen et al. 2009)

18 $$\frac{{c}_{g}(0)}{S{^{\prime}}_{gf}}={c}_{r}(0,t)$$

where ${\mathrm{S}}{^{\prime}}_{\text{gf}}$ is the partition coefficient of contaminants between GMB and fluid outside the GMB (Sangam and Rowe 2001); at present, there are relatively few studies on the value of ${\mathrm{S}}{^{\prime}}_{\text{gf}}$, which is usually considered to be equal to ${\mathrm{S}}_{\text{gf}}$(Xie et al. 2013).

The solution to Eq. (14) satisfying the boundary conditions Eqs. (15), (16), and (18) can be expressed as (Hahn and Özisik 2012)

19a $${c}_{g}(z)={\alpha }_{1}+{\beta }_{1}z$$

where ${\alpha }_{1}$ and ${\beta }_{1}$ are the constants to be solved based on the boundary conditions. Substituting the above equation into Eqs. (15) and (18), we are able to obtain ${\alpha }_{1}$ and ${\beta }_{1}$ as follows:

19b $${\alpha }_{1}={c}_{r}(0,t)S{^{\prime}}_{gf}$$

19c $${\beta }_{1}=\frac{{c}_{r}(0,t)S{^{\prime}}_{gf}-{c}_{0}{S}_{gf}}{{L}_{g}}$$

Substituting Eq. (19) into Eq. (16), we can obtain the functional relationship between ${f}_{r}$ and ${c}_{r}$

20a $${c}_{r}(0,t)={\eta }_{1}+{\eta }_{2}{f}_{r}(0,t)$$

and

20b $${\eta }_{1}=\frac{{c}_{0}{S}_{gf}}{S{^{\prime}}_{gf}}$$

20c $${\eta }_{2}=\frac{\theta {D}^{\prime}{L}_{g}}{{D}_{g}S{^{\prime}}_{gf}}$$

In summary, the boundary conditions for contaminant transport in cracks (Eq. 13) can be written as

21a $${c}_{r}(0,t)={\eta }_{1}+{\eta }_{2}{\left.\frac{\partial {c}_{r}(z,t)}{\partial z}\right|}_{z=0}$$

21b $${c}_{r}(\infty ,t)=0$$

21c $${c}_{r}(z,0)=0$$

Similarly, boundary conditions for contaminant transport in CCL (Eq. 11) are

22a $${c}_{r}^{\prime}(b,z,t)={c}_{r}(z,t)$$

22b $${c}_{r}^{\prime}(\infty ,z,t)=0$$

22c $${c}_{r}^{\prime}(x,z,0)=0$$

## Analytical solutions

### Steady-state solution

In the steady state, Eq. (13) describing the contaminant transport process in the crack can be simplified to the following equation:

23 $$-\frac{D}{{R}_{f}}\frac{{\partial }^{2}{c}_{r}(z)}{\partial {z}^{2}}+{\lambda }_{l}{c}_{r}(z)-\frac{\theta {D}^{^{\prime}}}{b{R}_{f}}{\left.\frac{\partial {c}_{r}^{^{\prime}}\left(x,z\right)}{\partial x}\right|}_{x=b}=0 0 \le z \le \infty$$

The boundary conditions are

24a $${c}_{r}(0)={\eta }_{1}+{\eta }_{2}{\left.\frac{\partial {c}_{r}\left(z\right)}{\partial z}\right|}_{z=0}$$

24b $${c}_{r}(\infty )=0$$

Equation (11) can be simplified to the following equation:

25 $$-\frac{{D}^{^{\prime}}}{{R}_{s}}\frac{{\partial }^{2}{c}_{r}^{^{\prime}}\left(x,z\right)}{\partial {x}^{2}}+{\lambda }_{s}{c}_{r}^{\prime}(x,z)=0 b \le x \le \infty$$

The boundary conditions are

26a $${c}_{r}^{\prime}(b,z)={c}_{r}(z)$$

26b $${c}_{r}^{\prime}(\infty ,z)=0$$

Equation (25) is a typical partial differential equation, and its solution should have the following form:

27 $${c}_{r}^{\prime}={\alpha }_{2}{e}^{-B{{\lambda }_{s}}^{1/2}(x-b)}+{\beta }_{2}{e}^{+B{{\lambda }_{s}}^{1/2}(x-b)}$$

and

28 $$B=(\frac{{R}_{s}}{D^{\prime}}{)}^{1/2}$$

The value of ${\alpha }_{2}$ and ${\beta }_{2}$ can be obtained by substituting the solution Eq. (27) into the boundary conditions Eq. (26) leading to

29 $${c}_{r}^{\prime}(x,z)={c}_{r}(z){e}^{-B{{\lambda }_{s}}^{1/2}(x-b)}$$

Substituting Eq. (29) into Eq. (23) and satisfying the boundary conditions required by Eq. (24); the transport solution equation of the contaminant in the crack can be obtained as

30 $${c}_{r}(z)=\frac{{\eta }_{1}}{1-g{\eta }_{2}}{e}^{gz}$$

and

31 $$g=-{[\frac{({\lambda }_{l}{R}_{f}b+\theta D^{\prime}B{{\lambda }_{s}}^{1/2})}{bD}]}^{1/2}$$

### Transient solution

For the coupled system consisting of Eqs. (11), (13), (21), and (22), the transient solution method will be shown below.

The Laplace transform of Eq. (11)

32 $$p{\overline{c} }_{r}^{\prime}=\frac{D^{\prime}}{{R}_{s}}\frac{{d}^{2}{\overline{c}}_{r}^{\prime}}{d{x}^{2}}-{\lambda }_{s}{\overline{c} }_{r}^{\prime}$$

where ${\overline{c} }_{\mathrm{r}}^{\prime}$ is the Laplace deformation of ${\mathrm{c}}_{\mathrm{r}}^{\prime}$.

33 $${\overline{c} }_{r}^{\prime}(x,z,p)={\int }_{0}^{\infty }\mathit{exp}(-pt){c}_{r}^{\prime}(x,z,p)dt$$

The solution is similar to Eq. (29), and the unique solution satisfying the boundary conditions takes the form

34 $${\overline{c} }_{r}^{\prime}={c}_{1}^{\prime}{e}^{-B{{P}_{s}}^{1/2}(x-b)}$$

35 $${P}_{s}=p+{\lambda }_{s}$$

In this equation, the constant ${\mathrm{c}}_{1}^{\prime}$ can be obtained using Eq. (22a) and after substituting into Eq. (34)

36 $${\overline{c} }_{r}^{\prime}={\overline{c} }_{r}{e}^{-B{{P}_{s}}^{1/2}(x-b)}$$

The derivative of ${\overline{c} }_{\mathrm{r}}^{\prime}$ at the intersection *x* = *b* is

37 $${\left.\frac{d{\overline{c} }_{r}^{\prime}}{dx}\right|}_{x = b}=-B{{P}_{s}}^{1/2}{\overline{c} }_{r}$$

Applying the Laplace transform to Eq. (13), we can obtain

38 $${p\overline{c} }_{r}+{\lambda }_{l}{\overline{c} }_{r}=\frac{\theta D^{\prime}}{b{R}_{f}}{\left.\frac{d{\overline{c} }_{r}^{\prime}}{dx}\right|}_{x=b}+\frac{D}{{R}_{f}}\frac{{d}^{2}{\overline{c} }_{r}}{d{z}^{2}}$$

Substituting Eq. (37) into Eq. (38)

39 $$\frac{{d}^{2}{\overline{c} }_{r}}{d{z}^{2}}-\frac{{R}_{f}}{D}({P}_{l}+\frac{{{P}_{s}}^{1/2}}{A}){\overline{c} }_{r}=0$$

and

40 $$A=\frac{b{R}_{f}}{\theta ({R}_{s}D^{\prime}{)}^{1/2}}$$

41 $${P}_{l}=p+{\lambda }_{l}$$

Equation (39) is a second-order ordinary differential equation, which has the general solution in the form

42 $${\overline{c} }_{r}={c}_{2}{e}^{z{r}_{+}}{+c}_{3}{e}^{z{r}_{-}}$$

where ${\mathrm{c}}_{2}$ and ${\mathrm{c}}_{3}$ are undetermined constants, and *r* has two forms

43 $${r}_{\pm }=\pm [{R}_{f}(\frac{{{P}_{s}}^{1/2}}{A}+{P}_{l})/D{]}^{1/2}$$

Since the solution value is finite, the first term in Eq. (42) must be eliminated, which means that ${\mathrm{c}}_{2}$ must be equal to 0. We get the remaining term, and Eq. (42) is transformed into

44 $${\overline{c} }_{r}={c}_{3}{e}^{-z[{R}_{f}(\frac{{{P}_{s}}^{1/2}}{A}+{P}_{l})/D{]}^{1/2}}$$

Using Eq. (21a), we are able to obtain ${\mathrm{c}}_{3}$ in the above equation. Applying the Laplace transform to Eq. (21a),

45 $${\overline{c} }_{r}(0,p)=\frac{{\eta }_{1}}{p}+{\eta }_{2}{\left.\frac{\partial {\overline{c} }_{r}\left(z,p\right)}{\partial z}\right|}_{z=0}$$

Substituting Eq. (44) into Eq. (45)

46 $${\overline{c} }_{r}(p)=\frac{{\eta }_{1}}{p\left(1+{\eta }_{2}\xi \right)}{e}^{-z\xi }$$

and

47 $$\xi =[{R}_{f}(\frac{{{P}_{s}}^{1/2}}{A}+{P}_{l})/D{]}^{1/2}$$

Using Stehfest’s numerical inversion method (Stehfest 1970a, b) for the Laplace inversion variation, we are able to obtain a semi-analytical solution of this equation

48 $${c}_{r}(t)=\frac{\mathit{ln}2}{t}{\sum }_{i=1}^{N}{V}_{i}{\overline{c} }_{r}(p)$$

and

49 $$p=\frac{i\mathit{ln}2}{t}$$

50 $${V}_{i}=(-1{)}^{\frac{N}{2}+i}\times {\sum }_{k=[\frac{i+1}{2}]}^{\mathit{min}(i,\frac{N}{2})}\frac{{k}^\frac{N}{2}(2k)!}{(\frac{N}{2}-k)!k!(k-1)!(i-k)!(2k-i)!}$$

where *N* is an even number. A better range of the values of *N* is from 10 to 14 (Lee et al. 1984). In this paper, *N* is taken as 10. Using Stehfest’s numerical inversion method, a semi-analytical solution of the concentration equation in the crack and CCL can be obtained.

The contaminant flux at any time in the crack at *z* = *L* can be obtained by the following equation:

51 $$J\left(L,t\right)=-nD{\left.\frac{\partial {\overline{c} }_{r}\left(z,t\right)}{\partial z}\right|}_{z=L}$$

## Validation of the proposed analytical solution

**Fig. 2:**
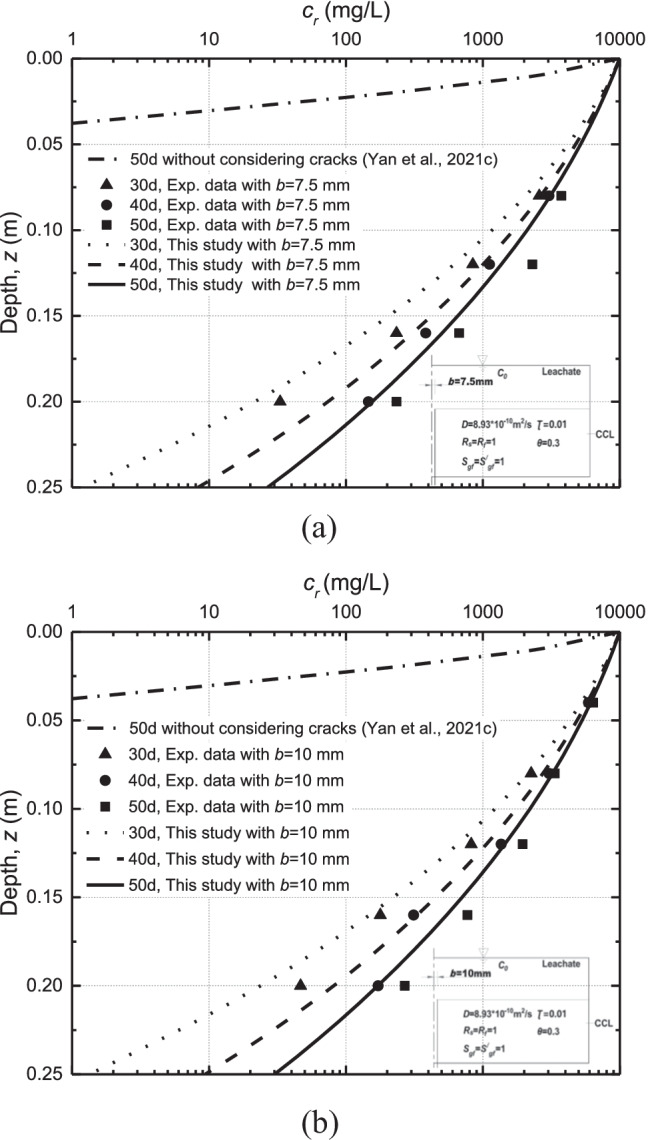
Comparisons of the results from this study and experimental data obtained by Li et al. (2018)

Experimental results from solute transport in CCL with a crack test was used to validate the proposed solution. The experiments carried out by Li et al. (2018) investigated the effect of cracks in CCL on the solute transport through the liner. The height of the soil column was 0.25 m. The concentration of contaminant (e.g., Cl^−^) at the top of CCL was fixed at 10,000 mg/L. The diffusion coefficient of Cl^−^ in CCL was reported to be 8.93 × 10^−10^ m^2^/s. Figures 2a and b show the spatial concentration of Cl^−^ under different crack thickness at 30 d, 40 d, and 50 d. The data reproduced from the literature is obtained by using the function of Digitizer in Origin Pro 2016. A good match between the analytical solution and experimental data can be found in Fig. 2. In this section, the coefficient of determination (*R*^2^) is used to show the validity of the present model. The *R*^2^ is given as

52 $${R}^{2}=1-\frac{{\sum }_{i=1}^{n}{\left({y}_{i}-{\widehat{y}}_{i}\right)}^{2}}{{\sum }_{i=1}^{n}{\left({y}_{i}-\overline{y }\right)}^{2}}$$

where *n* is the number of measurements, ${y}_{i}$ is the value of the *i*th observation in the experimental dataset, ${\widehat{y}}_{i}$ is the predicted value for the *i*th observation, and $\overline{y }$ is the average value of the validation dataset.

The *R*^2^ for the case with different crack widths and times is shown in Table 1. Generally, the present model shows a good prediction ability for the transport of contaminants in CCL. Additionally, the proposed analytical model shows a better performance for the case with larger crack width (see Table 1). It can be seen that the crack in CCL may significantly shorten the breakthrough of organic contaminants. For example, the maximum diffusion depth of contaminant (e.g., Cl^−^) is around 0.04 m in CCL without cracks at 50 days (Yan et al. 2021c). However, the concentration reaches 25 mg/L at the bottom of CCL for *b* = 7.5 mm at 50 days (Fig. 2a). The above results highlight the potential unpleasant breakthrough of contaminants in the liner barrier with the existence of cracks in the CCL.

**Table 1:** Values of *R*^2^

Crack width	Time (day)	Coefficient of determination, *R*^2^
*b* = 7.5 mm	30	0.92
	40	0.96
	50	0.83
*b* = 10 mm	30	0.96
	40	0.97
	50	0.94

## Results and discussions

Benzene was chosen to represent the contaminants in the landfill leachate. The thicknesses of the GMB and CCL are 1.5 mm and 0.75 m, respectively. The parameters used in this section are summarized in Table 2.

**Table 2:** Values of the parameters of GMB and CCL used in parametric studies

	GMB	CCL
Temperature (K)	298.15	
Atmospheric pressure (kPa)	100	
Thickness (m)	1.5 × 10^−3^	0.75
Diffusion coefficient (m^2^s^−1^)	${5} \, \times {1}0 ^{-{13}}$ ^d,f,g^	${8} \, \times\, {1}0^{-10}-8 \, \times \, {1}0^{-11}$ ^f^
Half-life of organic contaminants (years)	20^e^	20^e^
Porosity (-)	-	0.2–0.5^b^
Partition coefficients (-)	5^d,g^	-
Crack width (mm)	-	0–40^a,h^
Crack retardation factor (-)	-	1–19^c,i^

^a^Basnett and Brungard (1992). ^b^Musso et al. (2020). ^c^Tang et al. (1981). ^d^Touze-Foltz et al. (2012). ^e^ Wu et al. (2017). ^f^Xie et al. (2013). ^g^Xie et al. (2018). ^h^Xie et al. (2019). ^i^Yan et al. (2021a)

### Effect of crack width

**Fig. 3:**
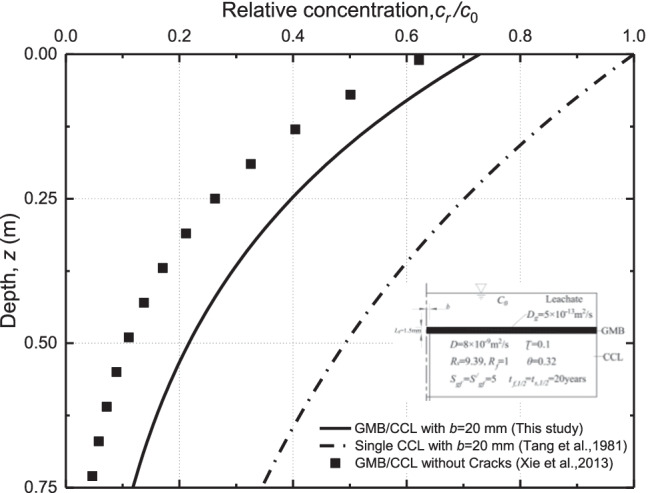
Longitudinal concentration distribution of organic contaminants in cracks under different scenarios at a steady state

The effect of crack on the steady-state concentration distribution of contaminants in the liner was investigated (see Fig. 3). The half-width of the crack (*b*) was 20 mm. The results of the present solution are compared to the results obtained by Tang et al. (1981) and Xie et al. (2013). The model developed by Tang et al. (1981) mainly focused on the contaminant transport in a single soil layer with a single crack. The study carried out by Xie et al. (2013) investigated the performance of GMB/CCL composite liner without considering the effect of cracks. Figure 3 shows the steady-state organic contaminant concentration distribution along with the depth for different scenarios. In order to highlight the differences among the different scenarios, the significance test was conducted by using the Kruskal–Wallis approach (Bougara et al. 2020). It is initially assumed that there is no significant difference among the three scenarios with a significance level of *α* = 0.05. The original hypothesis does not hold when *P* < *α*, which indicates a significant difference among the tests. A value of *P* = 1.06 × 10^−23^ is obtained for the three scenarios (e.g., the present study, Tang et al. 1981, and Xie et al. 2013), which demonstrates a significant difference among the cases.

As can be seen in Fig. 3, the crack existing in CCL can significantly affect the distribution of contaminant concentration for single CCL and GMB/CCL composite liner. For example, the relative concentration at the bottom of GMB/CCL composite liner for *b* = 20 mm is 3 times larger than that for the case without considering the crack. It is indicated that the GMB may provide a better diffusion barrier for the case without crack. Additionally, it can be seen that the relative concentration of contaminants at the surface of CCL is 1, 0.72, and 0.62 for the case with the single CCL, the composite GMB/CCL with and without crack, respectively. A smaller relative concentration profile is observed for the composite GMB/CCL compared to the single CCL due to the partitioning process of organic contaminants in GMB. The above results also indicated that the GMB layer is an effective barrier for the reduction of contaminants in the landfill liner system. The existence of cracks in the composite liner increases the concentration profile of the contaminant as the cracks play as preferential paths for the diffusion of the contaminant in the cracking GMB/CCL (Eq. 21). It is noted that the diffusion coefficient of contaminants in the crack is much larger than that in CCL, which results in a faster contaminant migration through the liner system and reduce the performance of GMB as a diffusion barrier.

The Monte Carlo method was adopted in this section to carry out the statistical analysis of different parameters (e.g., retardation factor, degradation of contaminants, the porosity of CCL, and crack width) (Jacoboni and Reggiani 1983). The ranges of the half-width of the crack (*b*), the crack retardation factor (*R*_*f*_), the half-life of organic contaminants in cracks (*t*_*f*, 1/2_), and the porosity of CCL (*θ*) was assumed to be 0–25 mm (Basnett and Brungard 1992; Xie et al. 2019), 1–19 (Tang et al. 1981; Yan et al. 2021a), 10–90 years (Yan et al. 2021a), 0.2–0.44 (Musso et al. 2020), respectively. The average values of these parameters *b*, *R*_*f*_, *t*_*f*,1/2_, *θ* are assumed to be 12.5 mm, 10, 50 years, 0.32, respectively, for the reference scenario. The above parameters are assumed to obey normal distribution. The 95% confidence interval of the reference scenario is constructed by a randomly generated 1000 dataset.

**Fig. 4:**
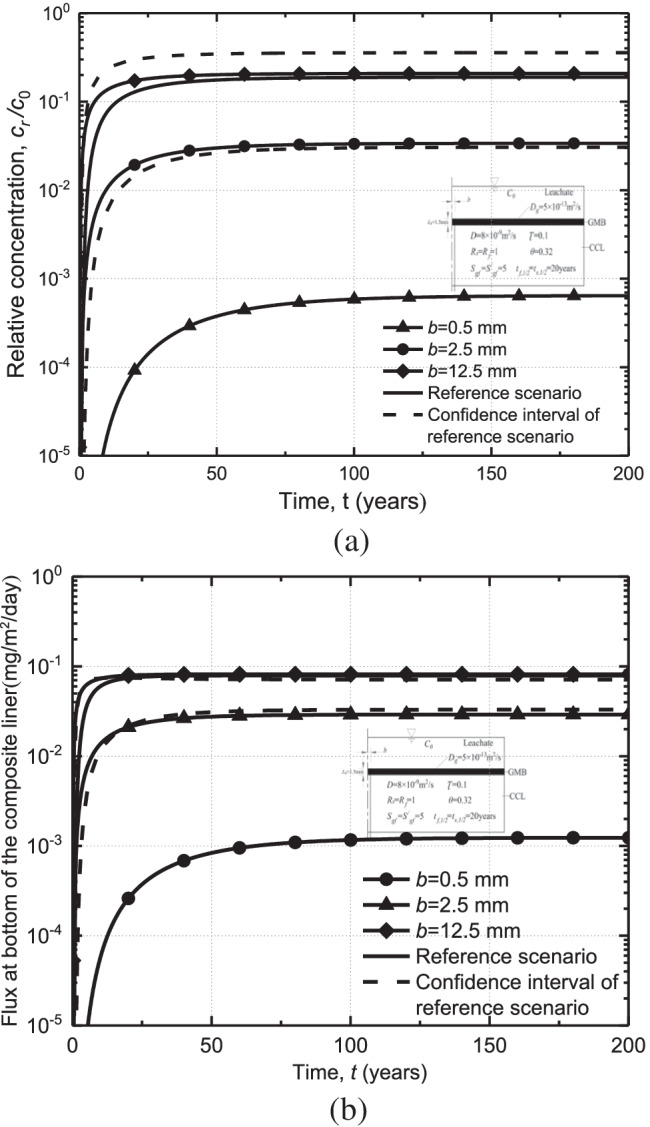
Effect of different crack widths on organic pollutant breakthrough curves and fluxes

Figure 4 shows the effect of crack width on the breakthrough curve (Fig. 4a) and flux (Fig. 4b) of organic contaminants at the bottom of the crack (*z* = 0.75 m). It can be seen that the crack width has a large effect on both the breakthrough time and bottom flux of organic contaminants. It should be noted that the value of relative concentration is shown by using logarithmic (log) scale as the value of relative concentration for the case with different crack widths varies a lot (e.g., 7 × 10^−4^ for *b* = 0.5 mm and 0.2 for *b* = 12.5 mm).

The breakthrough time is defined as the bottom concentration of contaminants reaching the maximum allowable concentration liner (Acar and Haider 1990; Zhan et al. 2017; Peng et al. 2020), which is assumed to be 10% *c*_0_ in this study. The breakthrough of the contaminant can be only found for the case with *b* = 12.5 mm (see Fig. 4a). It is indicated that the existence of cracks in CCL may damage the performance of the composite liner. Generally, the contaminant flux increases with the increase of crack width (Fig. 4b). For example, the steady-state fluxes for the case with *b* = 0.5 mm can be 90 times lower than that of *b* = 12.5 mm. Additionally, increasing the crack width can shorten the time required to reach a steady state. The time to reach a steady state is 150 a and 60 a for *b* = 0.5 mm and *b* = 2.5 mm, respectively. Additionally, the relative concentration and the bottom flux of *b* = 2.5 mm and *b* = 12.5 mm are almost within the 95% confidence interval of the reference scenario. The relative concentration and bottom flux dramatically decrease for a small width of crack (e.g.,$b\le 0.5$ mm). The above results indicated that considering the effects of the crack in CCL may help to design a more conservative, stable, and reliable landfill liner.

### Effects of degradation and adsorption

**Fig. 5:**
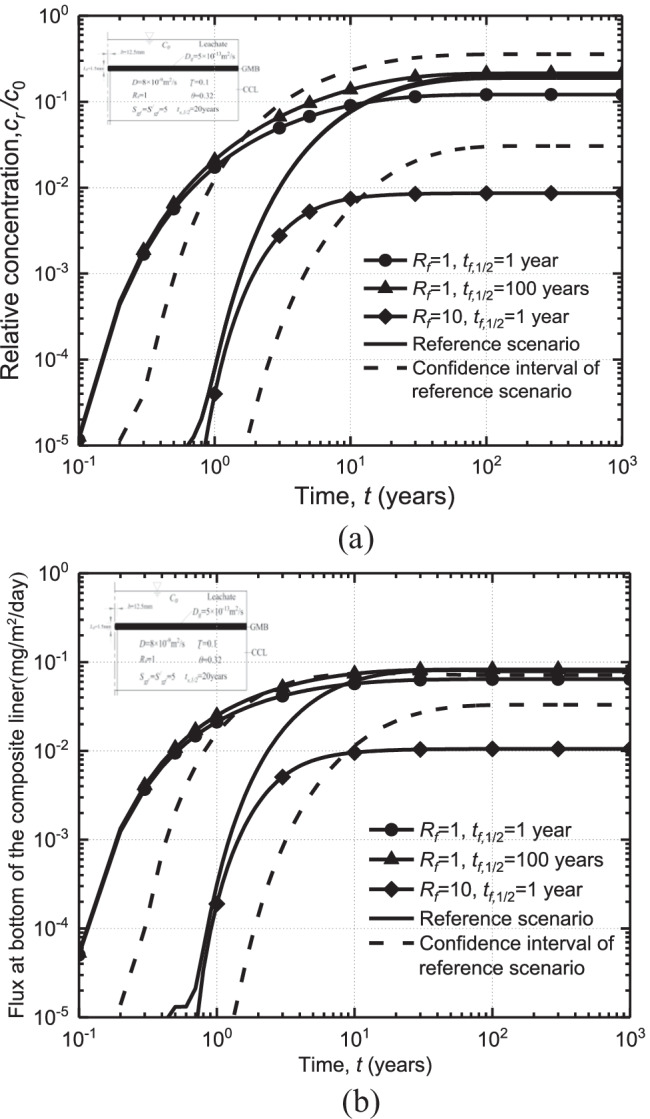
Effect of retardation factor and half-life of organic contaminants on the breakthrough curves and fluxes in the liner

Figure 5 shows the effects of half-life (${t}_{f,1/2}$) and adsorption retardation factor (${R}_{f}$) on organic contaminant migration in the liner system. Generally, increasing the adsorption factor and degradation rate of contaminants can effectively slow down the migration of contaminants in the liner system (Wood et al. 1990; Rowe et al. 2004; Xie et al. 2018). For example, increasing ${R}_{f}$ from 1 to 10 can lead to a reduction of steady-state bottom concentration of contaminants by a factor of 14 for ${t}_{f,1/2}$= 1 year. It is noted that the effect of adsorption is more significant than the degradation of contaminants on the breakthrough curve. For example, the steady-state concentration of contaminants for ${R}_{f}$= 1 can be 11 times larger than that for ${R}_{f}$= 10 with ${t}_{f,1/2}$= 1 year. At the same time, when ${R}_{f}$= 1, the steady-state concentration of ${t}_{f,1/2}$ = 100 years is only twice that of ${t}_{f,1/2}$ = 1 year. Increasing the retardation factor can significantly slow down the transport of contaminants, which may indirectly create a longer period for the degradation of contaminants.

### Effects of porosity of CCL and crack width

**Fig. 6:**
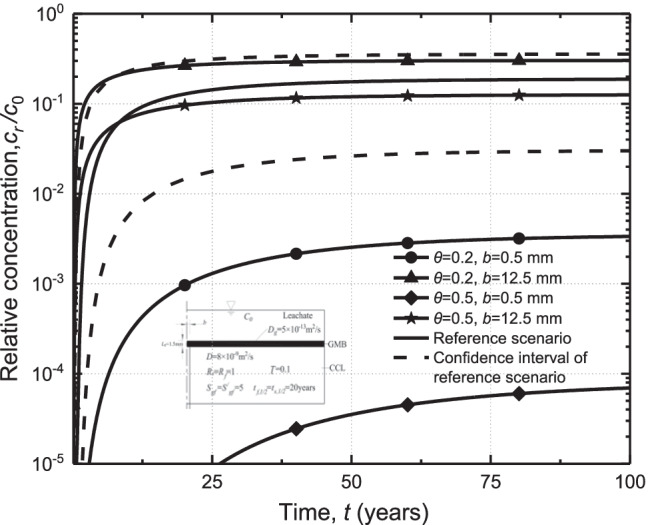
Breakthrough curves of organic contaminants in cracks with different widths and porosity

**Fig. 7:**
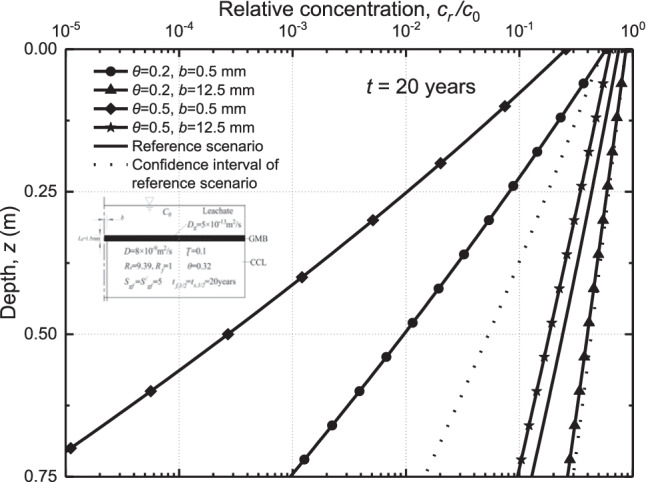
Effect of widths and porosity in cracks on organic contaminant concentrations at *t* = 20 years

The four scenarios, including scenario 1 (*θ* = 0.2, *b* = 0.5), scenario 2 (*θ* = 0.2, *b* = 12.5), scenario 3 (*θ* = 0.5, *b* = 0.5), and scenario 4 (*θ* = 0.5, *b* = 12.5) are used to investigate the relative sensitivity of the bottom relative concentration to the porosity of CCL and crack width (Fig. 6). It can be seen that decreasing the porosity of the CCL (i.e., scenario 1 and scenario 3) will enhance the transport of contaminants within the crack in the composite liner. When *b* = 0.5 mm, the maximum relative concentration for *θ* = 0.2 is about 3 × 10^−3^, which is around 40 times higher than that for *θ* = 0.5. This may be due to the reduction of the porosity of the CCL, which results in less contaminant diffusing into CCL. In order to represent the distribution of contaminants in cracks more clearly, the concentration of pollutants along cracks at *t* = 20 years was calculated (as shown in Fig. 7). When *b* = 0.5 mm, the relative concentrations of *θ* = 0.2 at *z* = 0.25 m, 0.5 m, and 0.75 m were 10 times, 33 times, and more than 100 times of *θ* = 0.5, respectively. It can be seen that the relative bottom concentration of organic contaminants for the case with *b* = 12.5 mm is within the 95% confidence interval of the reference scenario. However, decreasing the half-width of the crack from *b* = 12.5 mm to *b* = 0.5 mm results in a significant reduction of contaminant concentration. The variations of crack width play a more important role in contaminant transport compared to the changes of porosity. Additionally, the effects of CCL porosity on the breakthrough curve are more significant for the case with a thinner crack. For example, the steady-state concentration of contaminants for the case with *b* = 12.5 mm can be 100 and 1800 times larger than that of the case with *b* = 0.5 mm for *θ* = 0.2, and *θ* = 0.5, respectively. It is indicated that the variations of CCL porosity are dominant for the cases with the small cracks (e.g., *b* = 0.5 mm) as more amount of contaminant may diffusion into the matrix of CCL with a larger porosity. Interestingly, it can be found that there is a no-infiltration region for *θ* = 0.2. The phenomena can be explained by Fig. 7, which shows the contaminant concentration profile along with the depth at *t* = 20 years for different crack widths and porosity. It can be seen that the maximum penetration depth of the contaminants is 0.7 m for *θ* = 0.2 and *b* = 0.5 mm. This indicated that there is no contaminant reaching the bottom of the clay liner before 20 years and resulted in the no-infiltration region for the case.

## Limitations

The proposed analytical model was not without limitations. It is assumed that the concentration of contaminants is constant and variation of concentration is not considered for simplifying the calculation. However, the landfill leachate varies greatly at different ages, and the concentration of contaminants in landfill leachate may vary significantly with time. The leakage of the composite liner was not considered; this could be important for the cases with high leachate head and poor construction quality of geomembrane. Also, the case of multiple cracks affecting each other is not considered; the occurrence of cracks is not isolated and requires further study.

This study focuses on investigating the transport of contaminants in a GMB/CCL under constant temperature and pressure. The influence of non-isothermal diffusion (e.g., thermal diffusion and temperature-dependent diffusion coefficient) and consolidation of the liner under pressure is therefore not considered; however, previous studies indicated that leachate has a wide temperature range (Rowe and Hoor 2009; Bouazza et al. 2017; Yan et al. 2021c), and the bottom liner often deforms under high vertical stress (Yu et al. 2018; Rowe and Yu 2019). All these factors may affect the transport of contaminants in composite liner and should be further studied. Additionally, the first-order dynamic reaction model is adopted to investigate the degradation of organic contaminants in this study. It should be noted that the degradation of organic contaminants may show second-order or *n*th order degradation due to the influence of temperature and biological activities, although most organic contaminants can be described by the first-order biodegradation model (Báez et al. 2018; Shi et al. 2020). Other factors, such as heterogeneity and unsaturation of soil liner, may affect the performance of the liner system (Guyonnet et al. 2003; Yan et al. 2021c).

## Conclusions

An analytical model for the diffusion of organic contaminants in the GMB/CCL composite liner with CCL cracks is presented. Laplace transformation and Laplace inversion using the Stehfest method were adopted to derive the analytical solution. The proposed analytical solution is validated by a set of experimental data. The model was used to study parametrically the effects of the crack width, the porosity of CCL, degradation, and adsorption on the overall transport of organic contaminants in the cracked composite liner system.

It was shown that the cracks in CCL may shorten the breakthrough time and increase the bottom flux of organic contaminants. For example, increasing the crack width from 1 to 25 mm can result in a faster breakthrough of contaminants by a factor of 280. It is found that degradation of contaminants may play a more important role in the case with a larger retardation factor as increasing the retardation factor can slow down the transport of contaminants and indirectly create a longer period for the degradation of contaminants. Additionally, the reduction of porosity in CCL may enhance the transport of contaminants within the crack since the lateral diffusion of organic contaminants was greatly mitigated for the case with lower porosity.

## Data Availability

Data are available from the authors upon reasonable request.
